# Snakebites and Scorpion Stings in the Brazilian Amazon: Identifying Research Priorities for a Largely Neglected Problem

**DOI:** 10.1371/journal.pntd.0003701

**Published:** 2015-05-21

**Authors:** Fan Hui Wen, Wuelton Marcelo Monteiro, Ana Maria Moura da Silva, Denise V. Tambourgi, Iran Mendonça da Silva, Vanderson S. Sampaio, Maria Cristina dos Santos, Jacqueline Sachett, Luiz Carlos L. Ferreira, Jorge Kalil, Marcus Lacerda

**Affiliations:** 1 Instituto Butantan, Secretaria de Estado da Saúde de São Paulo, São Paulo, Brazil; 2 Departamento de Ensino e Pesquisa, Fundação de Medicina Tropical Doutor Heitor Vieira Dourado, Manaus, Brazil; 3 Escola Superior de Ciências da Saúde, Universidade do Estado do Amazonas, Manaus, Brazil; 4 Instituto de Ciências Biológicas, Universidade Federal do Amazonas, Manaus, Brazil; 5 Centro de Pesquisas Leônidas & Maria Deane (FIOCRUZ), Manaus, Brazil; Universidad de Costa Rica, COSTA RICA

Envenomings by snakebites and scorpion stings impose a high burden worldwide and result in considerable social and economic impact [1]. It is estimated that snakebite rates are as high as over 1.8 million cases per year, with associated deaths reaching more than 90,000 cases annually [2]. However, snakebites are a neglected condition with no associated WHO programmes for control and prevention. The countries most affected by snakebites are located in the intertropical zone in areas with high rates of field use for agriculture where the main affected populations are adult men working in agricultural activities [1]. Approximately two billion people are living in areas at risk for scorpion stings, with over one million accidents occurring annually worldwide [3]. However, the true burden of snakebites and scorpion stings is probably higher and difficult to estimate since only a few countries have a reliable system for epidemiological surveillance of these events.

In Brazil, the Ministry of Health implemented the National Program for Snakebites Control in 1986, extended to other poisonous animals in 1988. Since then, antivenom (AV) production has been standardized and all the AV production from the three national laboratories (Instituto Butantan, Fundação Ezequiel Dias, and Instituto Vital Brazil) was acquired by the Ministry of Health for distribution free of charge to patients. Five types of snake AVs are currently available: *Bothrops* AV (main one), *Crotalus* AV, *Bothrops-Crotalus* AV, *Bothrops-Lachesis* AV, and *Micrurus* AV. For scorpion stings, there are two types of AVs available in Brazil: *Tityus* scorpion AV and a polyvalent AV against *Loxosceles* and *Phoneutria* spiders and the *Tityus* scorpion. Table 1 summarizes information on snake and scorpion AVs produced in Brazil.

**Table 1:** Venom pools used for snake and scorpion AVs production in Brazil.

Type of AV	Venom	%
**Snake AVs**		
*Bothrops* AV	*Bothrops jararaca*	50.0
	*B*. *alternatus*	12.5
	*B*. *jararacussu*	12.5
	*B*. *moojeni*	12.5
	*B*. *neuweidi*	12.5
*Crotalus* AV	*Crotalus durissus terrificus*	50.0
	*C*. *d*. *collilineatus*	50.0
*Bothrops-Crotalus* AV	*Bothrops jararaca*	50.0
	*B*. *alternatus*	12.5
	*B*. *jararacussu*	12.5
	*B*. *moojeni*	12.5
	*B*. *neuweidi*	12.5
	*Crotalus durissus terrificus*	50.0
	*C*. *d*. *collilineatus*	50.0
*Bothrops-Lachesis* AV	*B*. *jararaca*	50.0
	*B*. *alternatus*	12.5
	*B*. *jararacussu*	12.5
	*B*. *moojeni*	12.5
	*B*. *neuweidi*	12.5
	*Lachesis muta*	100.0
*Micrurus* AV	*Micrurus corallinus*	50.0
	*Micrurus frontalis*	50.0
**Scorpion AVs**		
*Tityus* scorpion AV	*Tityus serrulatus*	100.0
*Loxosceles*-*Phoneutria* spiders and *Tityus* scorpion AV	*Loxosceles gaucho*	26.6
	*Phoneutria nigriventer*	20.0
	*Tityus serrulatus*	53.3

In 2013, 27,181 and 78,091 cases of snakebites and scorpion stings were reported by the Brazilian Ministry of Health, respectively [4]. The highest incidence was in the North region (52.6 cases/100,000 inhabitants) followed by the Midwest (16.4/100,000). These values, expected to be higher in remote areas of the Brazilian Amazon [5], may be underestimated due to considerable underreporting. Fig 1 presents the spatial distribution of snakebites and scorpion stings in the Brazilian Amazon.

**Fig 1:**
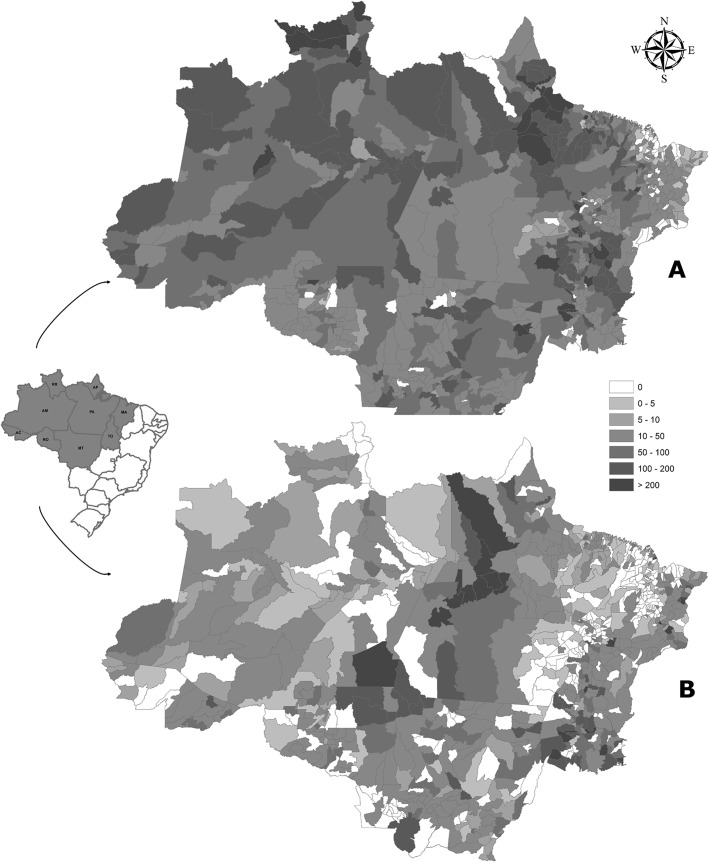
Spatial distribution of snakebites and scorpion stings in the Brazilian Amazon in 2013. Maps were created using incidence per 100,000 inhabitants. A) Snakebites are largely distributed in the Amazonian states, with several counties presenting incidences higher than 100 cases per 100,000 inhabitants, especially in Northern Roraima, Eastern Pará, and Amapá, and in unevenly distributed municipalities across all states. B) Large scorpion sting hot spots are observed in the Western Pará state and Southeastern Amazonas. Several other counties present incidences higher than 100 cases per 100,000 inhabitants in the states of Mato Grosso, Tocantins, and Maranhão.

## The Brazilian Amazon

Most of the information regarding snakebites in the Brazilian Amazon is based on surveillance data or hospital medical records. Community surveys were conducted with forest-dwelling Indigenous people and rubber tappers (*seringueiros*) [6], and inhabitants of ten riverine communities [7]. Snakebites predominate in adult males [7–12], strongly suggesting an occupational risk. People living in rural areas [8–11,13] and/or workers involved in farming, hunting, and forestry activities [6,7] were the most affected groups. Snakebite incidence correlated with the period of heaviest rainfalls [8,10–12]. Lower limbs were the most affected anatomical region. The time from the bite to the patient being seen at the hospital was usually longer than six hours [8,12,13]. Case fatality rates varied from 0.4% to 3.9% [6–8,10,11].

*Bothrops* snakebites are the most commonly recorded, accounting for around 80% of all cases [6,12,13]. *Lachesis* accidents predominated in the case series from Cruzeiro do Sul (Acre) (51.3%), followed by *Bothrops* and *Bothriopsis* (38%). However, the authors highlight a possible confounding factor in snake identification by the local population since both *Bothrops atrox* and *Lachesis muta* receive the same popular name “*surucucu*” in certain Amazonian areas [11].

Scorpion stings in the Amazon are mostly caused by *Tityus obscurus*, especially in the state of Pará, which can result in serious and fatal accidents [14,15]. A verbal autopsy study from the state of Acre found that 10% of tappers and 14% of Amerindians were stung by scorpions at least once in their lifetime [6].

**Fig 2:**
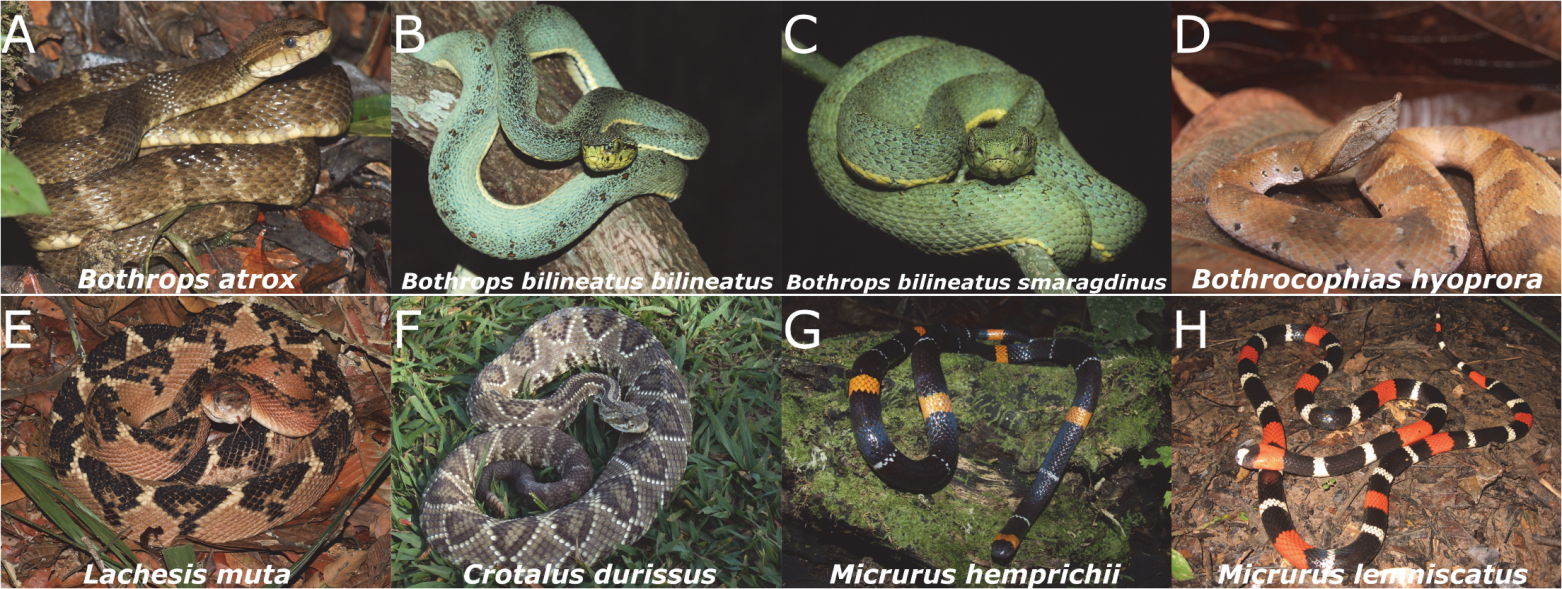
Snakes species involved in biting humans in the Brazilian Amazon. Pictures of the eight main snake species responsible for bites in the Brazilian Amazon region are shown (A–H). *Bothrops atrox* (A) is implicated in most of the human snakebites registered in the Brazilian Amazon region (80%–90%), followed by *Lachesis muta* (E).

**Fig 3:**
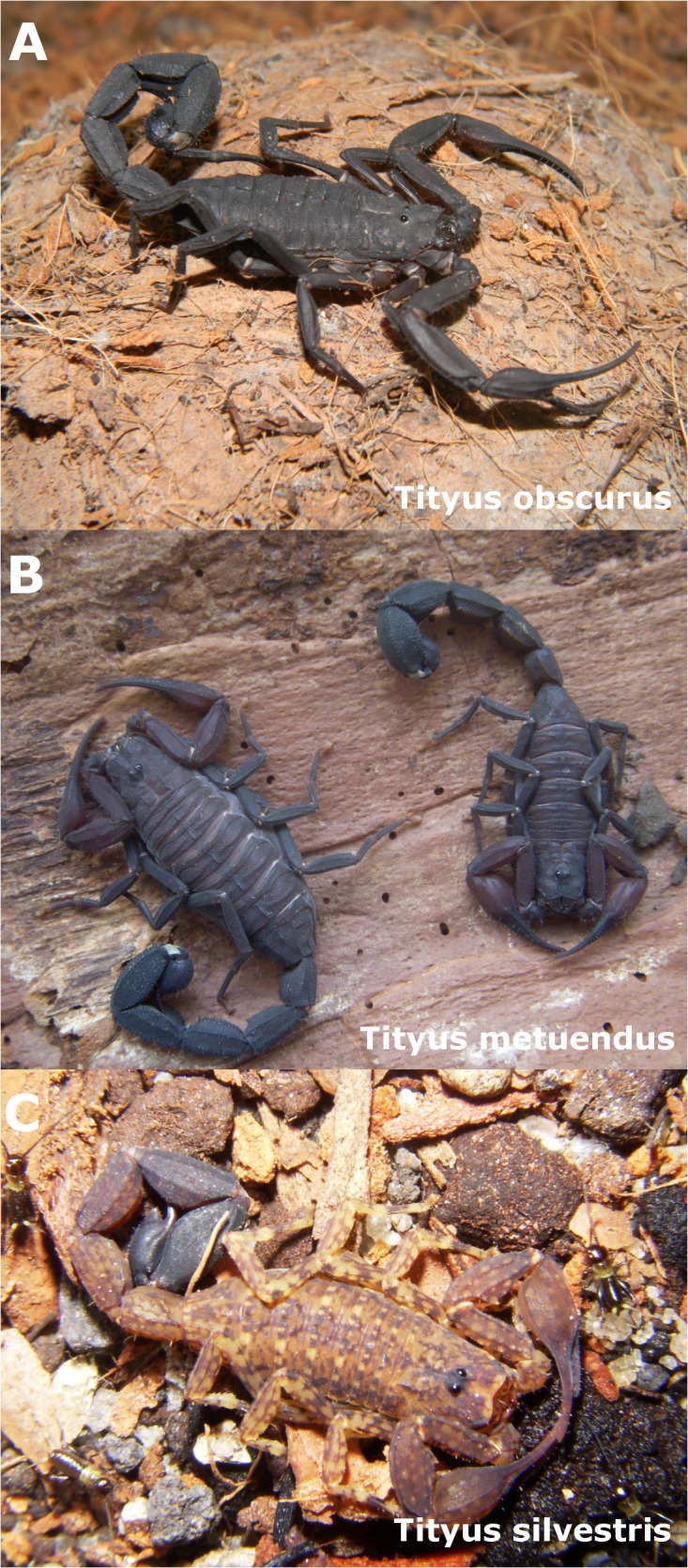
Main species of *Tityus* scorpions responsible for stings in the Brazilian Amazon. A) Female specimen of *Tityus obscurus*, the major species responsible for scorpion stings in the State of Pará. B) A male and a female *Tityus metuendus*, a species largely distributed in the Central and Western Amazon, especially in the Amazonas, Roraima, and Acre states. C) A male specimen of *Tityus silvestris*, a species found in the Southern Amazon.

Major species responsible for snakebites and scorpion stings in the Brazilian Amazon are presented in Figs 2 and 3, respectively.

## Research Gaps

Despite important efforts carried out during the past decades in Brazil to understand and control the problem of snakebite and scorpion sting envenomings, important gaps remain for the fulfillment of these goals, particularly in the Amazon region. A workshop was held in Manaus, Amazonas, in 2013 with representatives of Health Departments of Amazonian states, AV producers, universities, reference hospitals, and the Ministry of Health to identify research bottlenecks. A proposal to create the research network Snakebite and Scorpionism Network in the Amazon (*Rede de Ofidismo e Escorpionismo da Amazônia*-ROdA) emerged from researchers at the Butantan Institute and the Tropical Medicine Foundation Dr. Heitor Vieira Dourado.

The general aim was to enhance implementation of collaborative work and multicenter studies resulting in integration of services, research institutions, and health professionals. Identified research gaps are listed below.

### Burden on vulnerable populations

It is difficult for riverine and indigenous populations to reach health centers for treatment of venomous animal injuries. As a result, the number of cases detected officially is probably much lower than the real number. Current AVs require conservation in adequate facilities (2°–8°C), which are not always available in these remote settings. In addition, training of multidisciplinary teams is not always appropriate for indigenous health services regarding AV administration, side effect management, and case monitoring and surveillance.

Recommendations:

1. Assess disease burden through population- and hospital-based field studies in remote areas;
2. Seek innovation in the network for efficient distribution of immunobiologicals, especially interaction with other networks such as those providing vaccines;
3. Integrate different sectors (Health Surveillance Secretariat-SVS, Indigenous Health Special Secretariat-SESAI, National Agency of Sanitary Surveillance-ANVISA) for articulation of common strategies to be pursued with other ministries (Agriculture, Environment, Science, and Technology);
4. Review the skills of professionals assisting injured patients in areas without infrastructure and doctors according to international guidelines [16].

### Venom research and revision of the AV spectrum

Currently, AV immunoglobulins are the only treatment available for snakebites and scorpion stings envenomings. The WHO List of Essential Medicines includes them in the basic package of health care in affected countries. There is an urgent need to ensure availability of effective AVs and to improve their manufacture regulation. However, the possible interspecific venom variation associated with the geographical distribution of snakes may affect the effectiveness of therapeutic AVs against the Amazon *Bothrops* venom [17,18].

Current AV production methods, based on studies conducted in the 1980s, need updating in light of new technologies. Antivenom recommendations are based on experimental studies of cross-neutralization between specific venoms and AVs [19]. These excluded venom from *Bothrops atrox*, the main cause of snakebites in the Amazon. Efficacy of Brazilian AVs against venom from some Amazon *Bothrops* species has been investigated [20]. *Bothrops* AV showed neutralization of *B*. *atrox* venom major toxins [21,22]. Scorpion AV is produced by immunizing horses with *Tityus serrulatus* antigen, whereas *T*. *obscurus* is the most prevalent scorpion in the Amazonian region. However, some evidence suggests toxicity variation resulting from the diversity of *T*. *obscurus* venom in different Amazon areas [15]. Thus, new studies are needed and investment in technological development to assess different AV candidate formulations.

A major concern relates to the failure in AV distribution. Antivenoms are usually available in the municipal hospitals, as opposed to being distributed to peripheral health clinics. The lack of adequate cold chain impairs AV distribution to rural areas. Also, inadequate storage and transportation may result in loss of material. Freeze-dried AVs are available and one of the national producers (Butantan Institute) has been working to provide both liquid and freeze-dried products.

Recommendations:

1. Revise toxicity of snake venoms, including proteomics, as well as the potential for AV neutralization against major venom activities;
2. Study seroneutralization in experimental models to support the venom pool used to immunize animals for AV production, considering the absence of *Bothrops atrox* and *Tityus obscurus* venoms in these pools. Experimental studies should indicate the need for inclusion of new venoms, the new product needs to be validated by clinical and epidemiological data;
3. Perform stability studies of liquid AVs considering the Amazonian environmental conditions. Decisions on AV distribution, either liquid or freeze-dried products, should be based on careful and detailed analysis of the epidemiology of snakebites, the prevailing conditions, and health facilities available;
4. Study the mechanisms of venom action for different populations of snakes and scorpions in the Amazon (inter- and intra-species variations);
5. Study supporting action or herbal drugs with specific activity on certain venom components to enable complementary or alternative treatments.

### Priorities in clinical research

Although clinical research related to venomous animal injuries has increased, most publications are based on case reports and lack methodological rigor. Moreover, outcome definitions, such as severity ranking criteria, were empirically established, making the results even less generalizable. Clinical research from hospital-based studies (patient follow-up for evaluation of the frequency of events related to envenoming and their risk factors) and community observational studies (verbal autopsy studies and seroepidemiological surveys, group population cohorts, and qualitative studies) are needed.

**Fig 4:**
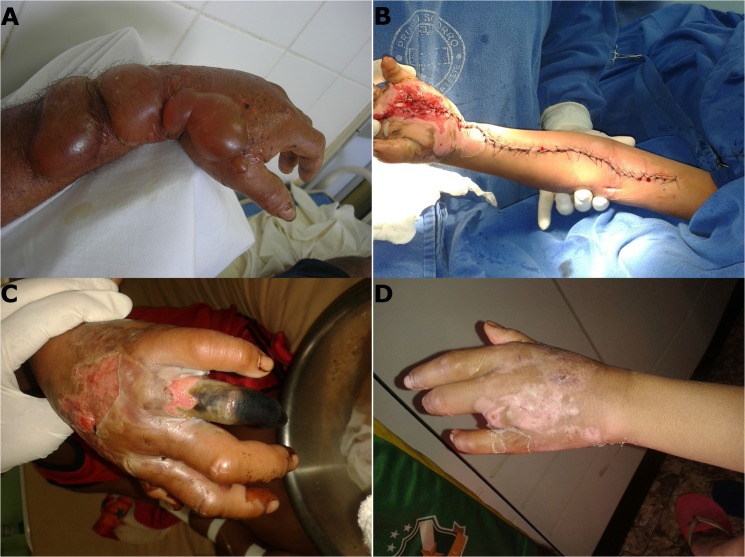
Local complications resulting from *Bothrops* snakebites. A) Envenoming on hand; this patient arrived 12 hours after the bite at the Hospital Municipal de Belterra, Pará, with swelling and serohaemorrhagic blisters on left upper limb and incoagulable blood. B) Severe envenoming on left hand; this patient arrived 24 hours after the bite at the Fundação de Medicina Tropical Dr. Heitor Vieira Dourado, Manaus, Amazonas, presenting compartmental syndrome in the left upper limb, requiring fasciotomy. C) Envenoming on left hand, patient arrived 24 hours after the bite at the Fundação de Medicina Tropical Dr. Heitor Vieira Dourado, Manaus, Amazonas, with an extensive area of edema and necrosis in the left upper limb and gangrene of the fourth finger. D) The same patient shown in C, after amputation of the fourth finger (in the healing phase).

Delays in patient care, along with the use of substances that may aggravate the conditions at the bite site, lead to a high frequency of local complications resulting from *Bothrops* (Fig 4) and *Lachesis* envenomings. However, severity is also possibly related to the composition of Amazon venoms [18]. Medical management of secondary infection, abscess, necrosis, and compartmental syndrome has been the subject of controversy, partly because of the lack of standardization regarding concepts and management protocols. The possibility of reducing local effects by means of drugs with anti-inflammatory activity, early antibiotic therapy for secondary infection, cross-neutralization of AVs for different types of accidents, and new complementary treatments need to be further investigated while observing good clinical practice and, preferably, in multicenter studies.

Systemic complications such as sepsis and acute kidney injury are less well known, and apparently less frequent, than local complications, but most frequent across the series from other regions in Brazil. The lack of patient follow up, including laboratory tests, appears to be related to this observation.

Recommendations:

1. Perform multicenter studies by standardization of clinical protocols for estimating independent risk factors for complications, also assessing AV efficacy (choice of outcomes) and defining objective criteria for recommending AV dosage;
2. Identification of the species responsible for snakebites in the Amazon requires the establishment of a gold standard method and determination of levels of antigenemia, preferably by means of rapid diagnostic tests;
3. Evaluate phase IV studies for adverse reactions (from three Brazilian manufacters) under AV pharmacovigilance;
4. Submit phase II/III protocols simutaneously to assess feasibility of comparative studies on efficacy and safety;
5. Plan training in Good Clinical Practices, as well as establish a link to the National Clinical Research Network (RNPC) from Teaching Hospitals;
6. Inform the regulatory agencies about the limitations and peculiarities of research involving animal envenomings, paying clarification and consultation to the National Human Reseach Ethics Council and the National Regulatory Agency.

### Adverse reactions and pharmacovigilance

Early adverse reactions (EAR) to AV therapy are expected events of varied frequency according to the type of AV used and individual hypersensitivity to heterologous proteins. Clinically, patients may present urticaria, itching, tachycardia, nausea, vomiting, abdominal colic, bronchospasm, hypotension, and angioedema. Over time, both the frequency and severity of early reactions have decreased due to the improvement of the AV purification process in Brazil. Frequency of delayed reactions (serum sickness) seems to be lower than EAR, but the true frequency is unknown. AV pharmacovigilance has been implemented, but reliable efficacy and safety data are still lacking.

There are no accurate predictive factors for side effects occurrence and preventing them is not always possible, even with the use of premedication containing corticosteroids and/or antihistamines. Existing studies do not include controls for the intervening variables and samples are of insufficient size, limiting the validity of the results [23].

Recommendations:

1. Perform multicenter phase IV studies, identifying sentinel hospitals for monitoring cases for both early and late reactions.

### Professional training

Despite the high incidence of injuries from venomous animals, there is a lack of systematic professional training on diagnosis, specific therapy, and clinical management of complications. Thus, AV misuse is not infrequent, either in quantity (number of ampoules administered) or the specific AV. Current training programs seek to link medical knowledge with the snakes’ and scorpions’ biology and surveillance. However, this approach often does not reflect the need for professional diagnosis algorithms and coherent and responsive medical management. Thus, adherence to medical training and courses in this area has been a major challenge. Furthermore, there is a high turnover of health professionals in small Amazon cities. Although communication technologies that greatly facilitate knowledge dissemination have proliferated in the area, these are still barely harnessed. The use of electronic media for training professionals in the management of envenomations is increasing and may be an alternative to classroom courses.

Recommendations:

1. Investments in training should cover all health professionals, including nurses who are critical to initial management of the patient and follow up of possible complications;
2. Update systematically all relevant diagnosis and treatment guidelines;
3. Encourage the use of technological resources for communication and other electronic media used in training programs and distance learning;
4. Include the topic in the undergraduate curriculum of health professionals with regionalized approaches to issues involving venomous animals;
5. Design new post-graduate and other courses, as well as interaction between graduate programs to increase the critical mass of professionals and researchers involved;
6. Inplement non-formal education activities for science communication, particularly aimed at school audiences.

### Fauna surveys and capture of animals for venom production

Traditionally, institutions producing AVs get animals caught from nature that are kept in vivariums and used to obtain venoms. However, environmental legislation restricts the collection and transport of wild animals. There is a requirement to establish specific policies to capture animals, and captive breeding is not done satisfactorily.

Field work is not limited to animal collection; it also includes studies of behavioral patterns such as diet, reproduction, and activities to establish phylogeny patterns for identifying risk factors of the envenomings. Increased knowledge on biodiversity of Amazon animals has applications on their use and species conservation. Zoological collections of invertebrates are also informative regarding their geographical distribution and diversity.

Recommendations:

1. Establish partnerships for capture of *Lachesis* snakes and transport to vivariums of laboratories producing AVs;
2. Guide *Lachesis* reproduction in captivity, which should be led by professionals familiar with their conditions both in nature and supportive maintenance environment;
3. Follow shared Standard Operating Procedures (SOPs) for the collections of venomous animals in order to facilitate exchange of information.

In addition to all the above recommendations, the group highlights the importance of international cooperative efforts towards the control of these neglected health problems through international partnerships, namely with other Amazonian countries.
